# 3D culture of cancer cells in alginate hydrogel beads as an effective technique for emergency cell storage and transportation in the pandemic era

**DOI:** 10.1111/jcmm.17078

**Published:** 2021-12-06

**Authors:** Batoul Alallam, May Kyaw Oo, Wisam Nabeel Ibrahim, Abd Almonem Doolaanea

**Affiliations:** ^1^ Department of Pharmaceutical Technology Faculty of Pharmacy International Islamic University Malaysia Kuantan Malaysia; ^2^ Department of Biomedical Sciences College of Health Sciences QU Health Qatar University Doha Qatar; ^3^ Biomedical and Pharmaceutical Research Unit QU Health Qatar University Doha Qatar; ^4^ IKOP Sdn Bhd Faculty of Pharmacy International Islamic University Malaysia Kuantan Malaysia

## Abstract

Due to the restrictions in accessing research laboratories and the challenges in providing proper storage and transportation of cells during the COVID‐19 pandemic, having an effective and feasible mean to solve these challenges would be of immense help. Therefore, we developed a 3D culture setting of cancer cells using alginate beads and tested its effectiveness in different storage and transportation conditions. The viability and proliferation of cancer cells were assessed using trypan blue staining and quantitative CCK‐8 kit, respectively. The developed beads allowed cancer cells survival up to 4 weeks with less frequent maintenance measures such as change of the culture media or subculture of cells. In addition, the recovery of cancer cells and proliferation pattern were significantly faster with better outcomes in the developed 3D alginate beads compared to the standard cryopreservation of cells or the 2D culture conditions. The 3D alginate beads also supported the viability of cells while the shipment at room temperature for a duration of up to 5 days with no humidity or CO_2_ support. Therefore, 3D culture in alginate beads can be used to store or ship biological cells with ease at room temperature with minimal preparations.

## 1 INTRODUCTION

With continuously changing situations because of COVID‐19, lockdowns are suddenly enforced. Researchers, especially in low‐ and middle‐income countries, face new limitations due to the short time allowed to work in the laboratories and the congested use of equipment. For example, in Malaysia, we are currently under the third general lockdown. The decisions of the lockdown (general or localized) were announced at very short notice that severely affected ongoing laboratory experiments. Many researchers faced the problems when handling cell cultures with significant loss due to the inability of maintaining the cells. In general, standard culture measures of cancer cells require changing the culture media 2–3 times per week in most cases. If the researcher is not able to maintain the cells, then cryopreservation is the available option to store the cells, or the researcher might choose to shift his laboratory work to another facility or institute, which can handle the cells. However, with the short notice given before enforcing the complete lockdown, the cell culture laboratories become congested with insufficient time to cryopreserve the cells or prepare them for shipping, which usually needs special reagents for cryopreservation and shipping in dry ice.^1^ Therefore, it is mandatory to establish an affordable and easy methodology that could help in overcoming these challenges.

Here, we responded to these two problems by applying 3D cell culture technique in a new scope of application. We tested 3D cell culture as a method for maintaining cells for up to 4 weeks with minimal laboratory work needed. We also applied this method for shipping the cells at room temperature with no special preparations or precautions.

We used three human cancer cell lines, HepG2 hepatocellular cancer, A549 lung cancer and U2OS osteosarcoma cells (American Type Culture Collection), originally cultured using common culture method in T25/T75 flasks (referred here as 2D cell culture). HepG2, A549 and U2OS cells were maintained in standard DMEM medium, F‐12K and McCoy's 5A media, respectively, all supplemented with 10% FBS and incubated at 37°C and 5% CO_2_.

The preparation of the 3D cell culture included reagents such as 1% w/v sodium alginate (pharmaceutical grade IL‐6G, Kimica) and 1% w/v calcium chloride (Merck). The 3D alginate hydrogel beads were prepared by a gentle ionotropic gelation method^2^ (Figure S1). For more practicability and reduced use of equipment, all the solutions were sterilized using 0.22 µm sterile filters. The formed 3D alginate beads were transferred into T25 flask loaded with 25 ml media and maintained at 37°C and 5% CO_2_ for 4 weeks with media change once every week. The flasks were observed regularly under the inverted microscope (Mshot). All experiments in this study were conducted in triplicates for validation of the results. During the first 8 days, the cells were tested for cell viability using semi‐quantitative trypan blue assay method and for proliferation potential using the quantitative colourimetric Cell Counting Kit 8 (CCK‐8)^3^ (Supplementary Information S1) and compared the results with a control group of 2D culture. After 4 weeks, the cells were recovered from the beads by dissolving the beads rapidly in citrate buffer pH 6.0 (Supplementary Information S1). The recovered cells were then cultured in T25 flask and observed for cell attachment after 1 h, 2 days and 6 days.

**FIGURE 1:**
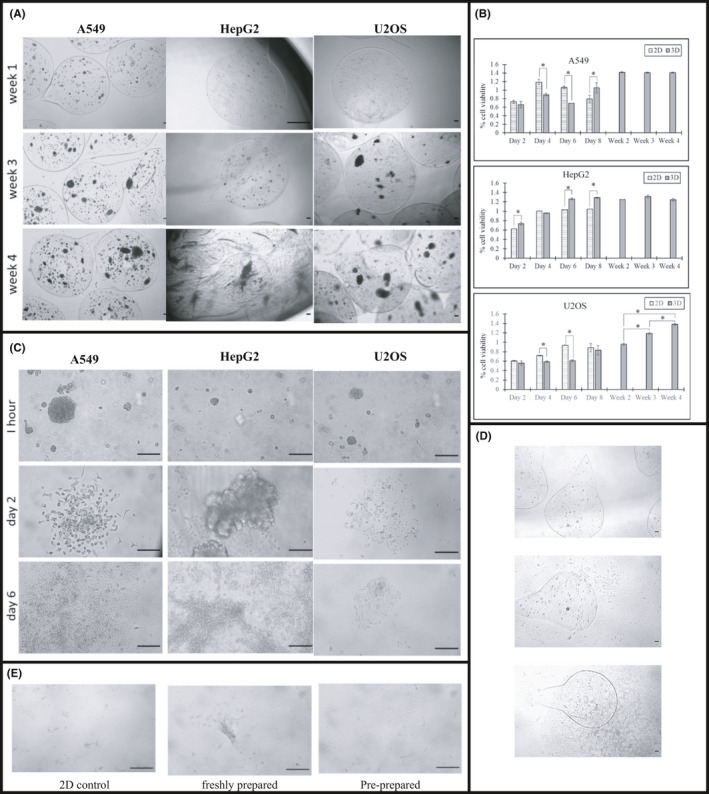
(A) 3D alginate beads culture of A549, HepG2 and U2OS cells up to 4 weeks with media change once per week, 4× objective, scale bare is 500 µm. (B) Cell viability of 2D and 3D cell culture in alginate beads over 8 days, *p*‐values <0.05 are given asterisks and considered significant. (C) A549, HepG2 and U2OS cells recovered from 4‐week culture in 3D alginate beads (media change once per week) then cultured as 2D and imaged after 1 h, 2 days and 6 days, 20× objective, scale bar is 100 µm. (D) A549 cells cultured in 3D alginate beads for 5 weeks without media change, showing different levels of cell leakage outside the beads, 10× objective, scale bar is 500 µm. (E) A549 cells recovered after shipping, 10× objective, scale bar is 500 µm

The average size of the alginate beads ranged from 6.8 to 8.0 mm. The cells continued to grow inside the beads forming spheroids. The spheroid size increased significantly until week 4. After 2, 3 and 4 weeks the spheroids of A549 reached a diameter of 20–65, 28–65 and 99−187 µm, respectively (Figure 1a, Figure S2). HepG2 and U2OS cells did not form spheroids until week 3 where their sizes were 14 and 40 µm, then increased at week 4 to 87–204 and 112–160 µm, respectively (Figure 1a).

The average cell viability estimated by trypan blue assay of A549, HepG2 and U2OS was more than 85% (Table S1). Using the quantitative CCK‐8 assay, we found that the cell proliferation of A549 and U2OS cells was slightly lower than that of the control 2D culture until day 6; however, it increased at day 8 (Figure 1b). HepG2 cell viability was higher than that of the 2D culture throughout the 8 days. The cell viability of 2D cell culture did not proceed after 8 days due to the overgrowth of the cells in multilayer and clear cell death manifested as large number of floating cells. There was no significant decrease in cell viability in 3D cultures from week 1 to week 4. All three cell lines were able to attach and proliferate after recovery from 4‐week cultures and HepG2 cells exhibited the best growth (Figure 1c, Figure S3).

The results above proved the practicability of 3D cell culture in alginate beads as a tool to maintain the cells and recover them with minimal laboratory work and equipment use.

In a separate more extreme experiment, A549 cells were prepared in 3D alginate beads and cultured at 37°C and 5% CO_2_ for five weeks in complete media within T25 flasks without any change of media throughout the duration. After the 5‐week culture, the beads showed different levels of cell leakage. The leaked cells continued to grow normally as 2D cell culture (Figure 1d). After that, we recovered the cells and cultured them successfully in 2D cell culture. These results are promising and may help researchers amid the COVID‐19 pandemic. With no need to change media for up to 5 weeks, it is feasible to quickly convert the ongoing 2D cell culture that is prone to abandonment due to a sudden lockdown into 3D cell culture using alginate beads. After five weeks, the cells can be recovered again to grow as 2D cell culture. It is worth to be mentioned that the recovered cells in this method resume the growth at faster rates compared to cryopreservation. For example, HepG2 took approximately 2 days to attach and proliferate after recovery from 5‐week beads, while it took two weeks to show good attachment and proliferation after recovery from liquid nitrogen storage.

Finally, to test the suitability of the 3D cell culture in alginate beads for cell shipping at room temperature, we used freshly prepared and pre‐prepared beads of A549, pre‐incubated in T25 flasks at 37°C and 5% CO_2_ for 1 and 7 days, respectively. 2D cell cultures were shipped along with the 3D culture setting as a control. Similar flasks were also kept in the incubator as controls. The flasks were shipped from our laboratory in Kuantan, Malaysia to another laboratory in Kuala Terengganu, Malaysia (about 250 km distance) then returned back in the same shipment over a period of 5 days. The flasks were shipped following a general parcel shipping procedure using local courier without any additional precautions or instructions. To record the condition during the shipping, we assembled a data logger using Arduino Nano, DHT11 sensor, piezo vibration sensor and SD card module (Figure S4). The data logger recorded the temperature, relative humidity and vibration every 1 s. The temperature ranged between 30 and 35°C with few hours of excursion to 40°C (Figure S5). The relative humidity ranged mostly between 50% and 70%. The vibration sensor showed low vibration during the shipping with short time excursion to high vibration concomitant with the temperature increase to 40°C, which is expected to be the latest step in the delivery to the target laboratory.

Upon receiving the flasks back to our laboratories, the cells were recovered and checked for viability and attachment. Majority of the cells shipped in the 2D culture arrived dead with no recovery in standard culture conditions to resume normal growth. On the other hand, the viability of cells recovered from the shipped pre‐prepared and freshly prepared beads was 74.9% and 60.0%, respectively, and 81.7% and 85.3%, respectively, in the incubator control flasks. The longer the cells were incubated before shipment the more viable they were after shipping. This observation might be attributed to the ability of the cells to adapt with 3D cell culture conditions during the 1‐week incubation. Nevertheless, in both cases, we were able to recover the cells from the beads and resume growth in 2D cell culture (Figure 1e, Figure S6). These results provide sufficient evidence for the efficiency of alginate 3D cancer cells beads as medium for storage and shipping of viable and active cancer cells without the need to cryopreserve the cells or provide the basic requirement of culture environment at room temperature. It can be used for freshly prepared beads (for emergency shipping for example) or for pre‐prepared beads (up to 1 week in the incubator in this study).

## 2 CONCLUSION

3D cell culture in alginate beads can be used to maintain living cells for up to 4 weeks with minimal support in situations like restricted access to the research laboratories. It can also be used as feasible and effective mean to transport cell lines between research laboratories that might need up to 5 days of transport conditions without additional preparations. This novel application provides an affordable and easy method for the preservation of biological cells and requires less frequent maintenance practices such as the need to subculture the cells or change the media. These promising findings are supported by the better recovery rates from 3D beads compared to 2D culture setting and cryopreserved cells.

## CONFLICT OF INTEREST

The authors confirm that there are no conflicts of interest.

## AUTHOR CONTRIBUTION

**Batoul Alallam:** Data curation (equal); Formal analysis (equal); Investigation (equal); Methodology (equal); Writing – original draft (equal). **May Kyaw Oo:** Validation (equal). **Wisam Nabeel Ibrahim:** Supervision (equal); Validation (equal); Visualization (equal); Writing – review & editing (equal). **Abd Almonem Doolaanea:** Conceptualization (equal); Project administration (equal); Supervision (equal); Validation (equal); Visualization (equal); Writing – review & editing (equal).

## Supporting information

Supplementary Material

## Data Availability

The data that supports the findings of this study are available in the supplementary material of this article.
