# Supplementary material for: 3D culture of cancer cells in alginate hydrogel beads as an effective technique for emergency cell storage and transportation in the pandemic era
Source: J Cell Mol Med. 2021 Dec 6;26(1):235–8. doi: 10.1111/jcmm.17078 (PMC8742195; doi:10.1111/jcmm.17078)
Supplement: Supplementary file 1 — Supplementary Material [file JCMM-26-235-s001.docx]

**SUPPLEMENTARY INFORMATION**

**METHODS**

**Preparation of 3D alginate beads**

The cells were harvested from T25 flasks at confluency <60%, and resuspended in fresh media at a cell density of 1×10^5^ cells/mL. Total of 10^5^ cells/mL were well-suspended in the alginate solution at 1:10 volume ratio and aspirated into a 5-mL syringe attached to 21G needle (Fig. 1). The syringe was held in an upright position (90°) over 9 cm petri dish containing 20 mL of calcium chloride crosslinking solution and positioning the tip of the needle 3-4 cm above the liquid surface. The cell suspension was dropped into the petri dish at about one drop per 10 seconds. Then, the dish was closed and left for curing at room temperature for 10 min. Calcium chloride solution was aspirated by gently pipetting. Then, 5 mL of fresh complete media were added to the petri dish and let stand at room temperature for 15 min. Then the media were replenished and new 25 mL fresh media were dispensed onto the formed 3D cell beads.

**Cell viability assay using trypan blue**

An aliquot of 100 μL from the cell suspension was taken and mixed with 100 μL of trypan blue solution. The mixture was incubated at room temperature for 3 minutes to allow trypan blue to penetrate to the dead cells. 10 μL of the cell suspension was loaded into hemocytometer and the cells were counted under MF53 inverted microscope (Mshot, China). The total number of dead/live cells and the percentage of viability were evaluated by two operators (hereafter mentioned as observer1 and observer2).

**Cell viability using Cell Counting Kit 8 (CCK-8)**

Cell viability was assessed for cell metabolic activity using Cell Counting Kit-8 (CCK-8, DOJINDO, Japan). This assay allows continuous monitoring of the cell viability without the need to kill the cells like in the case of MTT assay. To assess the cell viability, flasks of 3D cells were prepared as it is described before. As a control, flasks of 2D cells were prepared by seeding at an equal density of 3D cell beads and incubated overnight. After one day, 100 *µ*L of CCK-8 reagent was added to each flask and the flasks were incubated at 37 °C and 5% CO_2_. At predetermined time points, the optical density of 1 mL of each flask medium was recorded using Tecan Infinite 200 microplate reader at 450 nm. The percent of cell viability values of 3D cells were calculated after blank subtraction and compared to 2D counterpart at each time point.

**
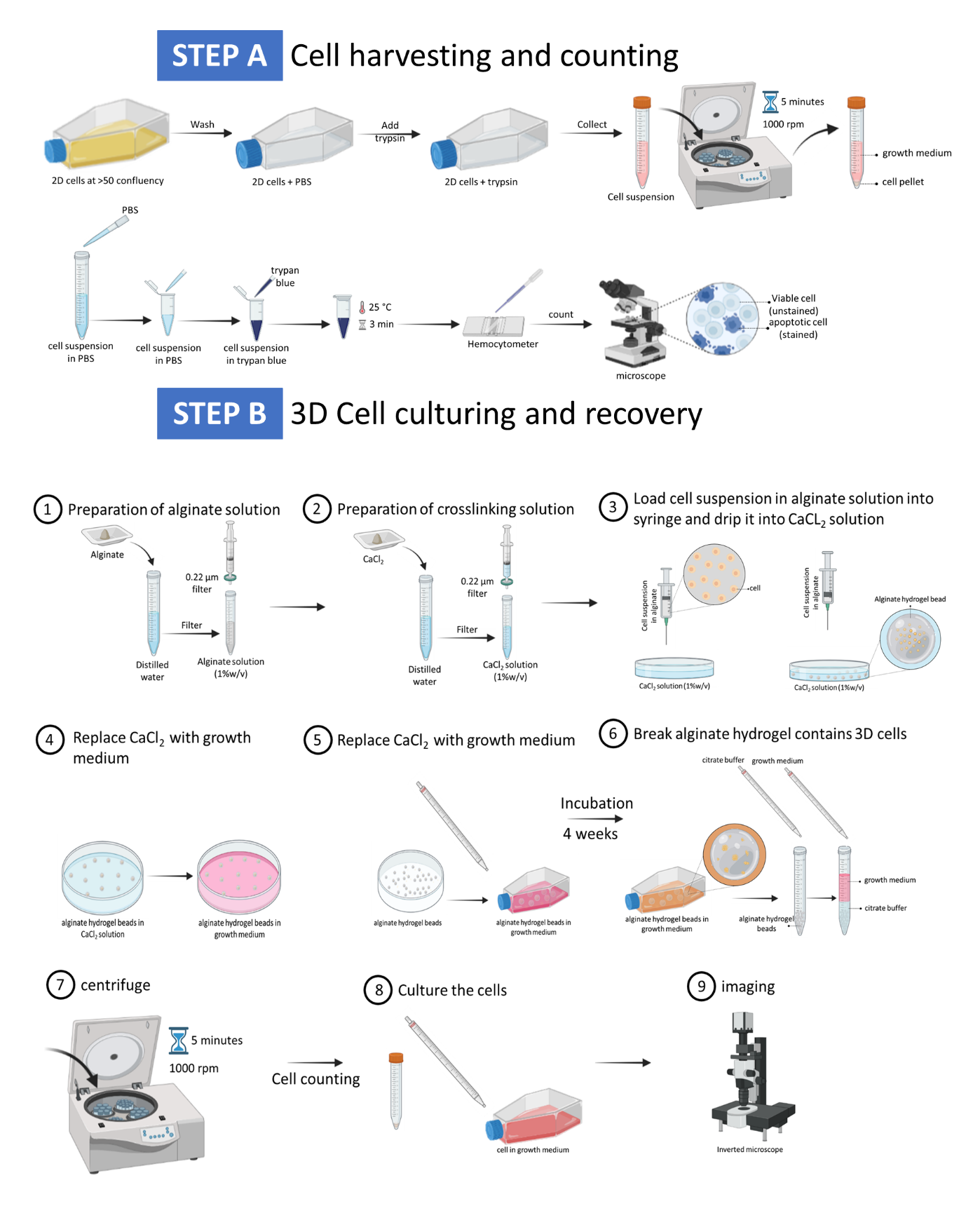
**

**Fig. S1: Schematic experimental flow for the preparation and recovery of 3D cell culture in alginate hydrogel beads.**

**Cell recovery from the 3D alginate beads**

In each flask containing 3D alginate cell culture beads, the medium was discarded and the 3D alginate hydrogel beads were moved into 15 mL falcon tube. 1 mL of filter-sterilized citrate buffer (0.05 M, pH 6.0) was added to the beads and incubated for 10 seconds at room temperature to dissolve the beads, then 10 mL of the media was added. The cells were centrifuged at 1000 rpm for 5 min, and the cell pellet was resuspended in 1 mL media then cultured in T25 flask incubated at 37 °C and 5% CO_2_.

To prepare the citrate buffer: Dissolve 1.285 g of sodium citrate dihydrate and 0.121 g of citric acid in 80 mL of distilled water o the solution. Adjust the solution to pH 6.0 using HCl or NaOH.

Add distilled water to make up the volume 100 mL. Sterilize the solution by passing it through 0.22 µm sterile filter.

**Table S1: Cell viability estimated by trypan blue assay of A549, HepG2, and U2OS after 8 days culture in 3D alginate beads.**

|  | Observer1 | | | Observer2 | | |  |
| --- | --- | --- | --- | --- | --- | --- | --- |
| Cells | mean | STD | CV (%) | mean | STD | CV (%) | P value |
| A549 | 92.01 | 2.58 | 0.028076 | 93.12 | 3.23 | 0.034668 | 0.184 |
| HepG2 | 87.89 | 1.56 | 0.017712 | 90.46 | 1.58 | 0.017457 | 0.483 |
| U2OS | 90.25 | 2.64 | 0.029238 | 90.51 | 3.10 | 0.034267 | 0.168 |

| **a)** |
| --- |
| 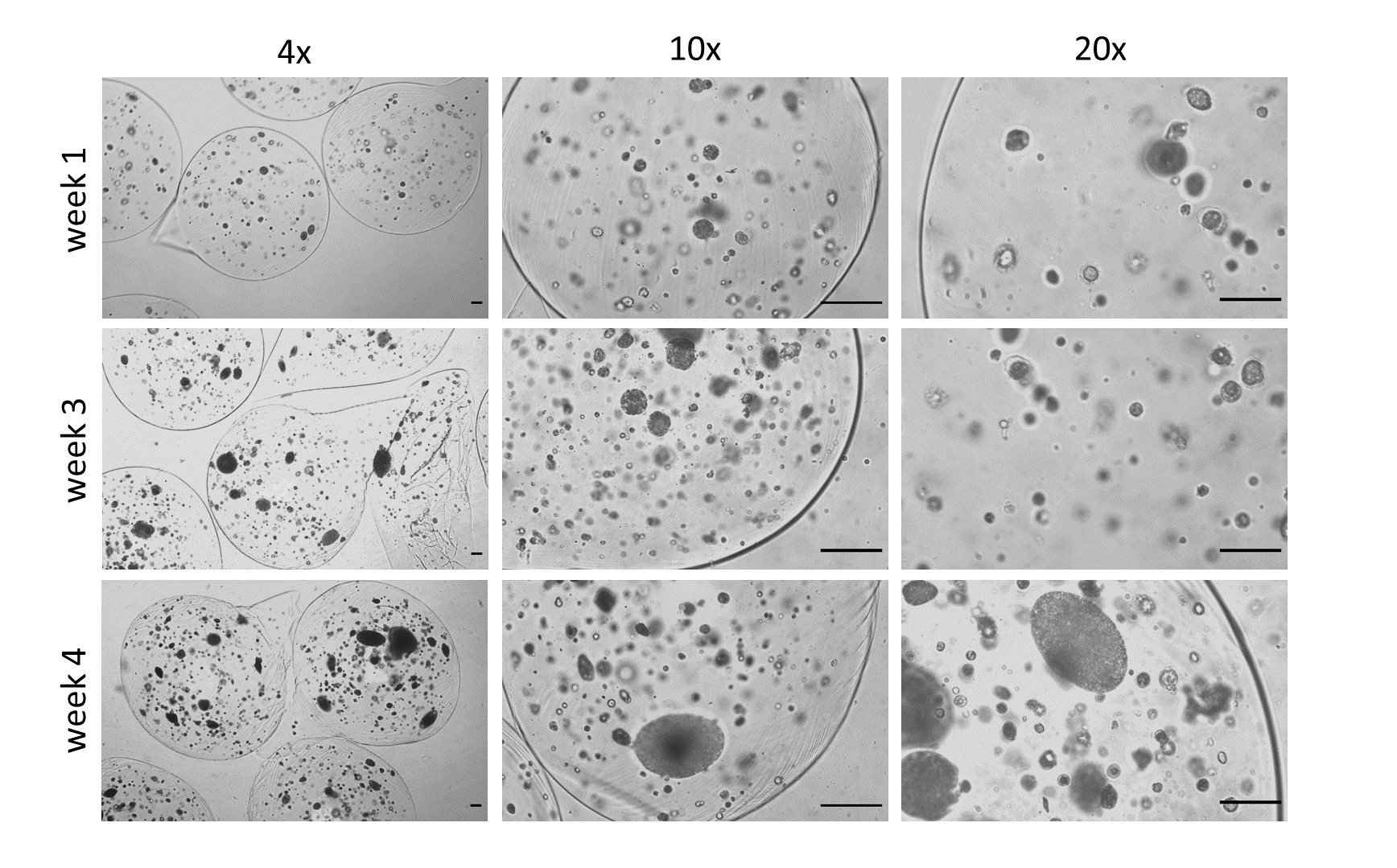 |
| **b)** |
| 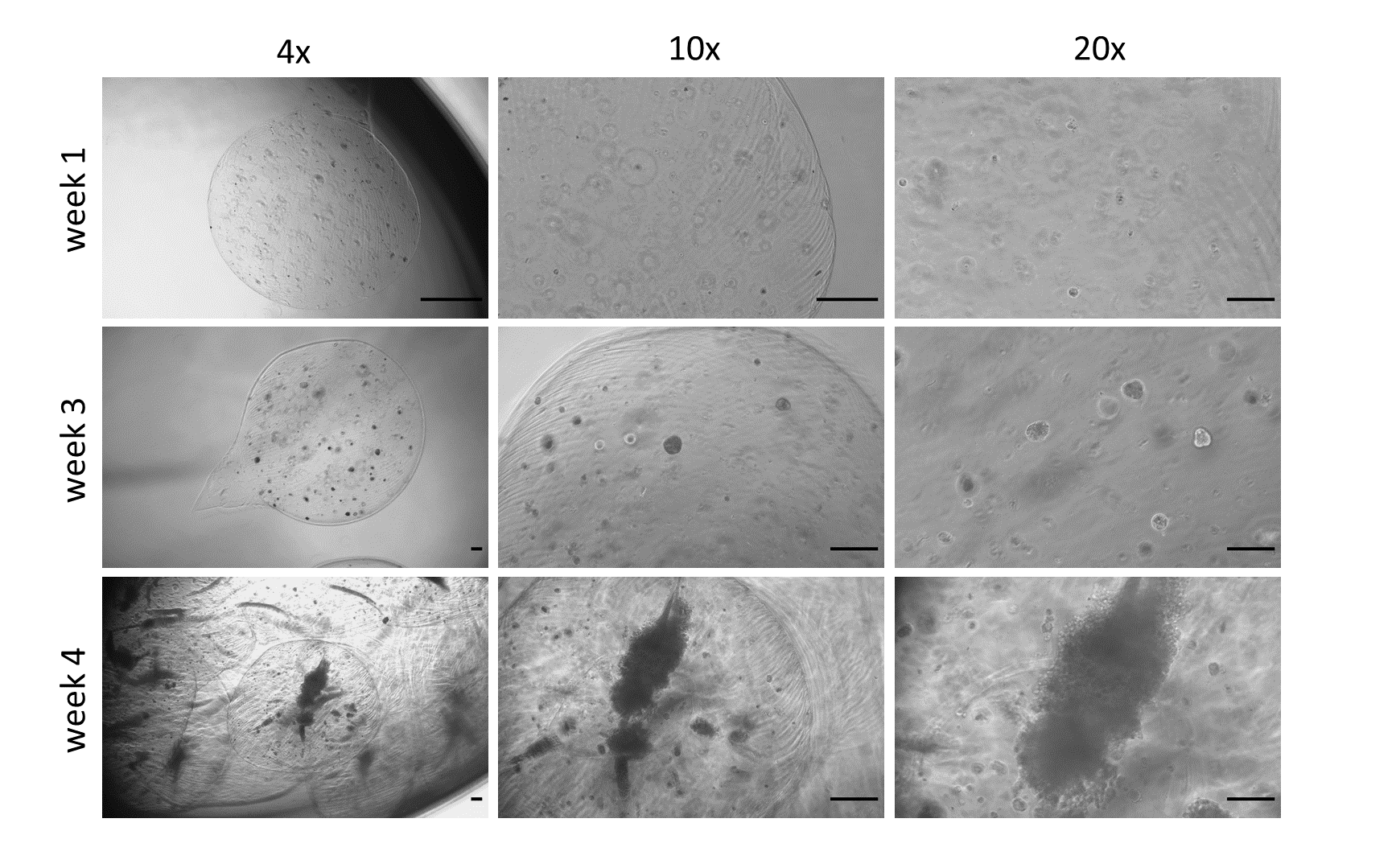 |
| **c)** |
| **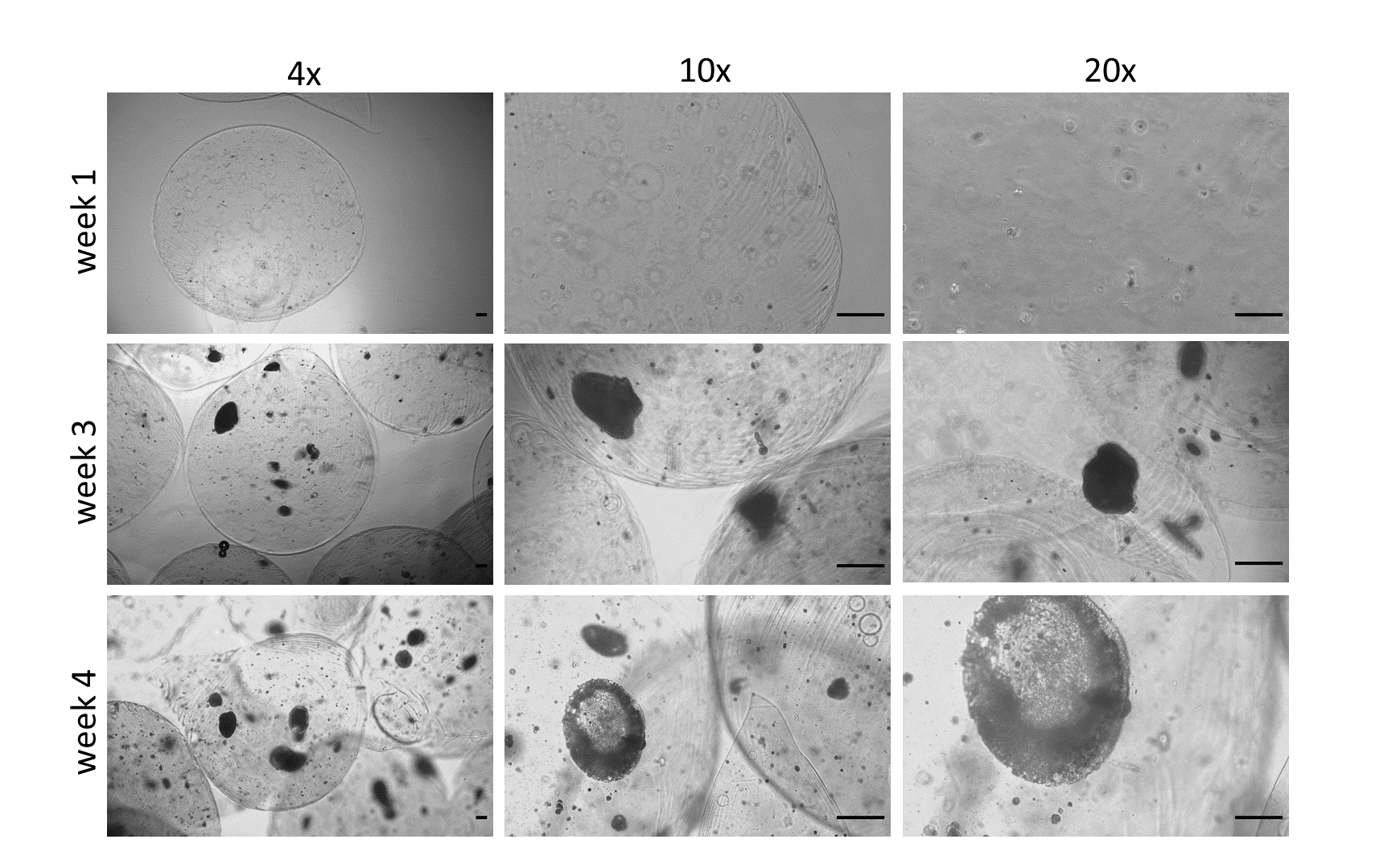** |
| **Fig. S2: Alginate hydrogel contains a) A549, b) HepG2 and c) U2OS cells. Scale bare for 4x magnification is 500 µm and for 10x, and 20x is 100 µm.** |
|  |

| **a)** |
| --- |
| 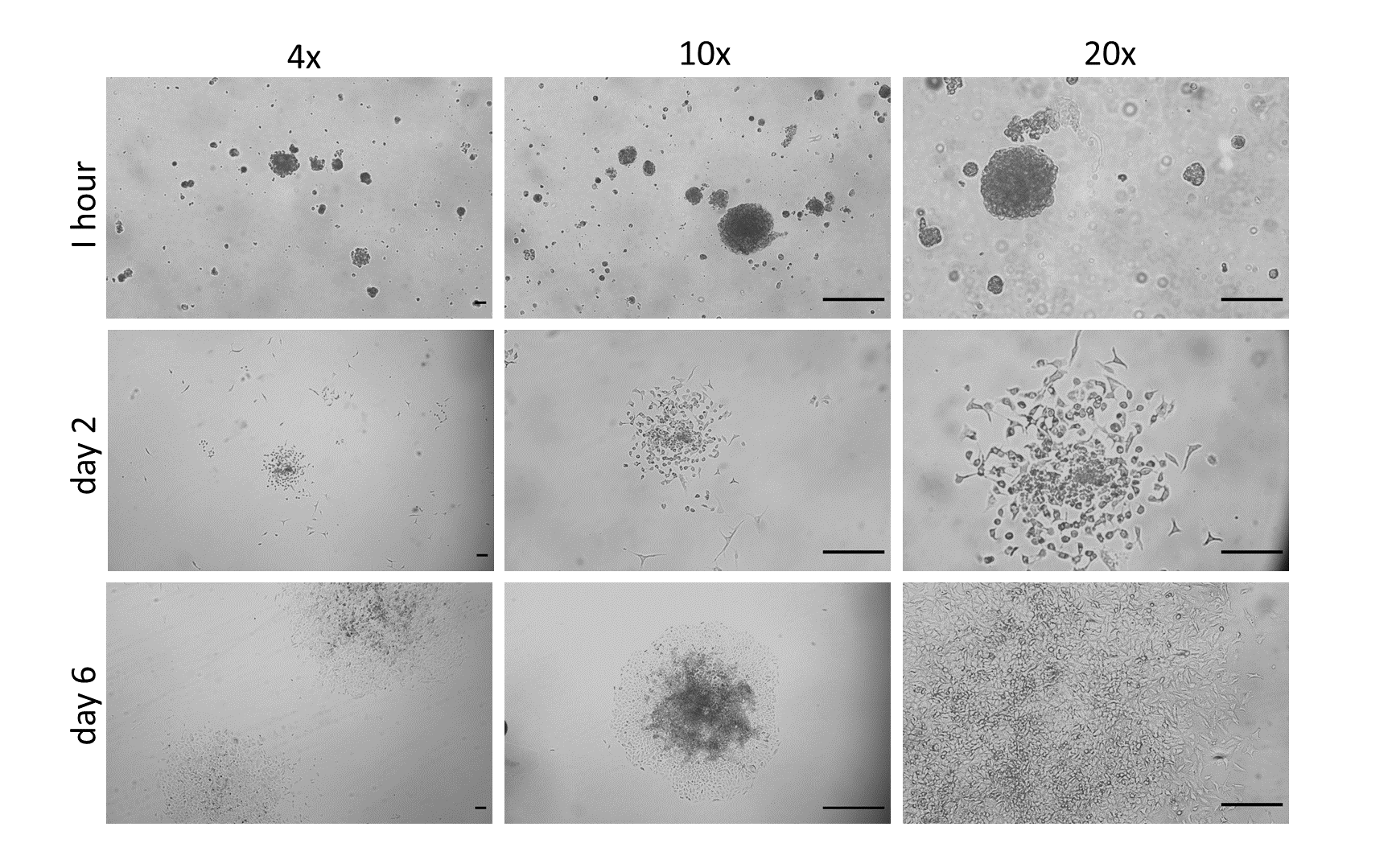 |
| **b)** |
| 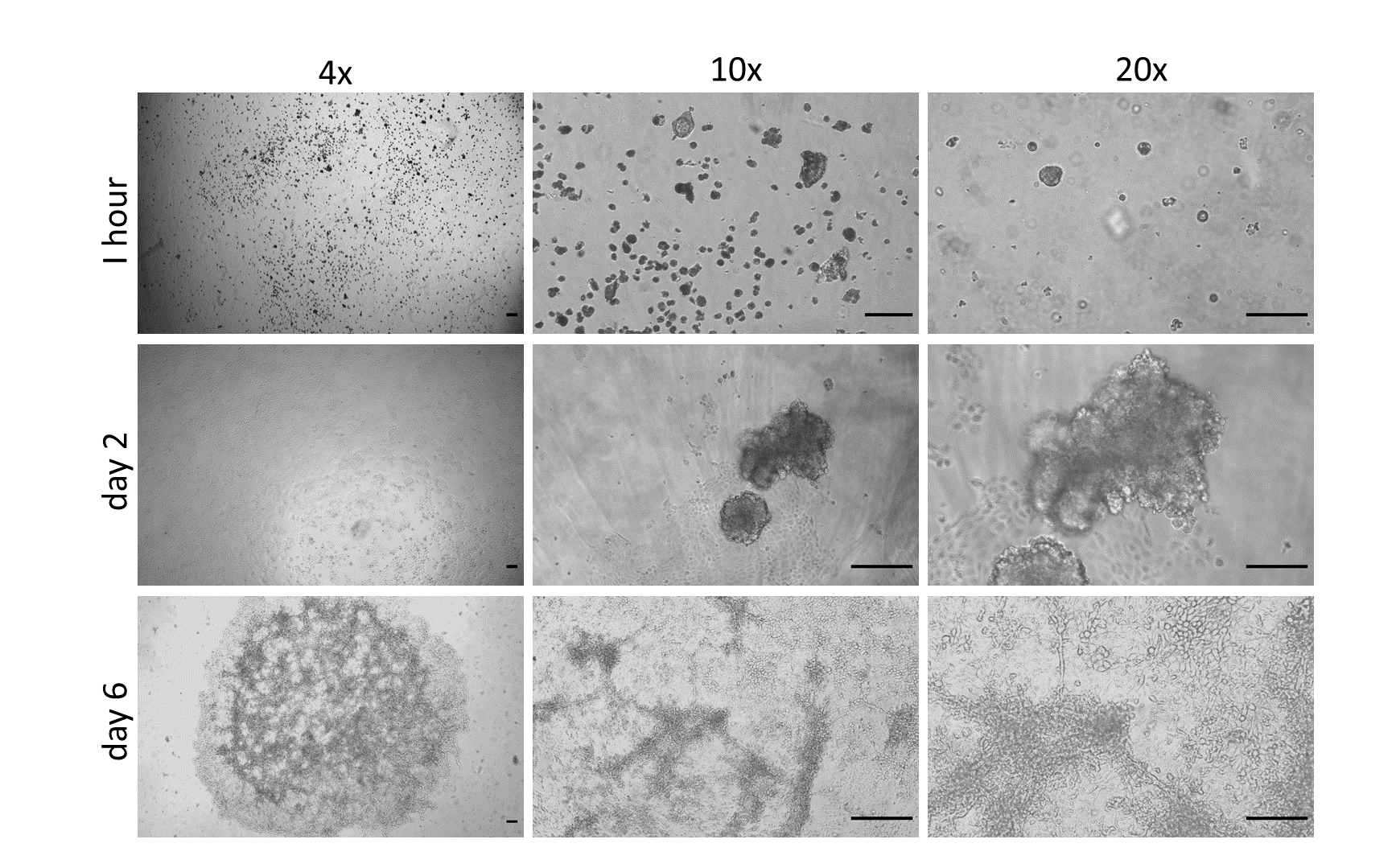 |
| **c)** |
| 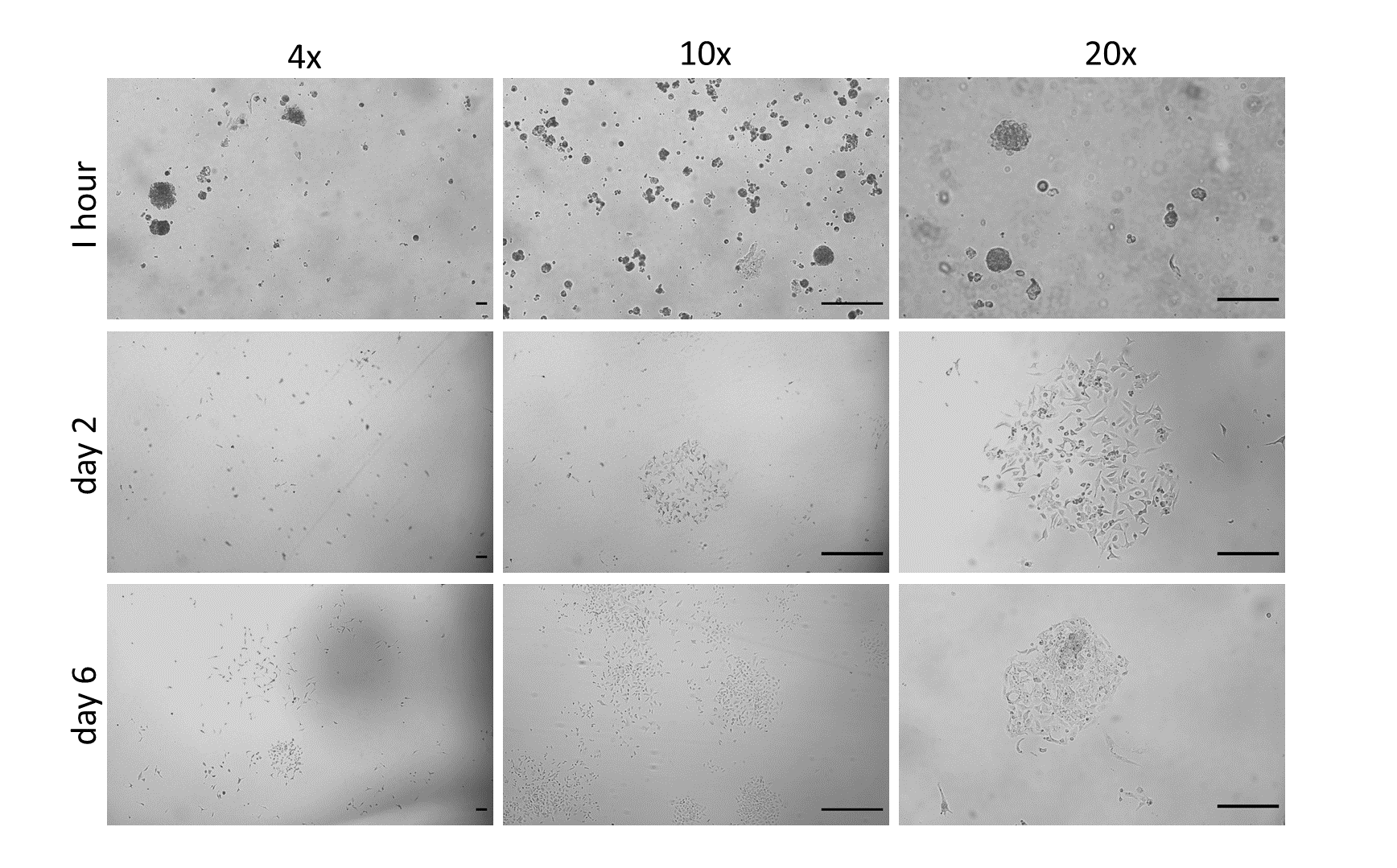 |
| **Fig. S3: spheroids of a) A549, b) HepG2 and c) U2OS cells after 1 hour, 2 days and 6 days of recovery from alginate hydrogel beads. Scale bare for 4x magnification is 500 µm and for 10x, and 20x is 100 µm.** |

**Arduino code and design of the data logger**

| **a)** | **b)** |
| --- | --- |
| 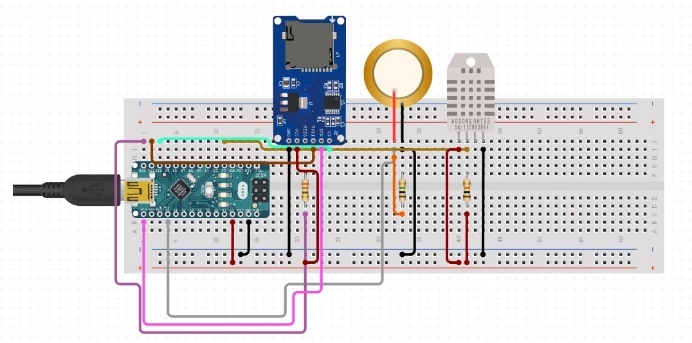 | 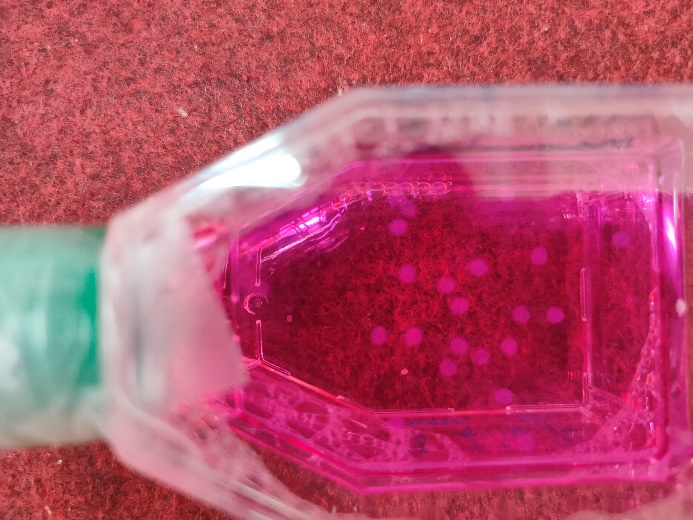 |
| **c)** | **d)** |
| 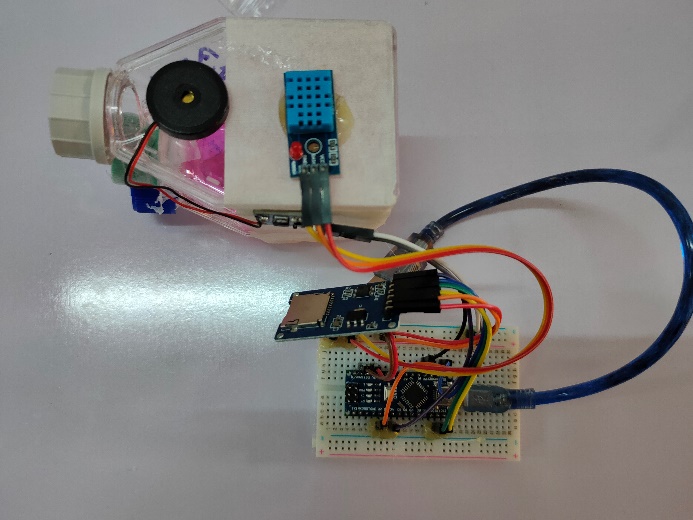 **DHT11 temperature and humidity sensor**  **Vibration sensor**  **SD card**  **Arduino nano** | 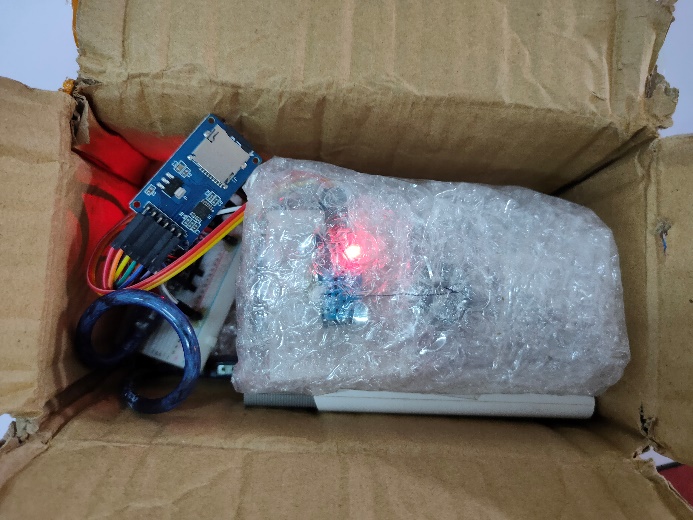 |
| **Fig. S4: Assembled data logger: a) schematic circuit to show the parts. Actual parts may differ. Pin connection may slightly differ, b) 3D beads inside the T25 flask, c) Actual picture of the set up, d) Actual shipment.** | |

| **a** |
| --- |
| 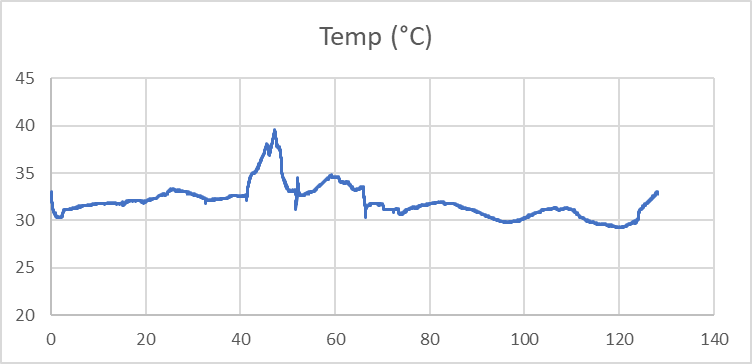  Time (h) |
| **b** |
| 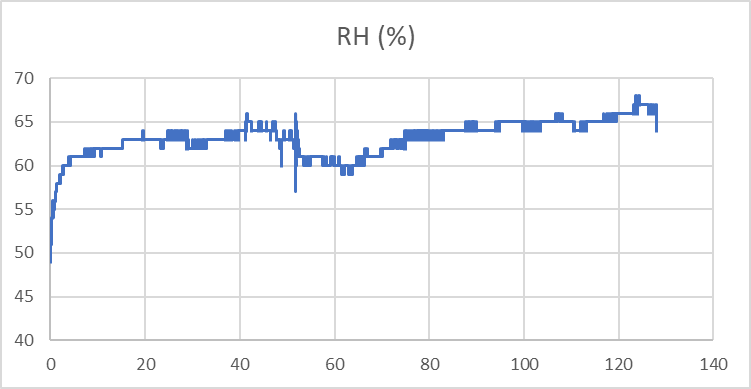  Time (h) |
| **c** |
| 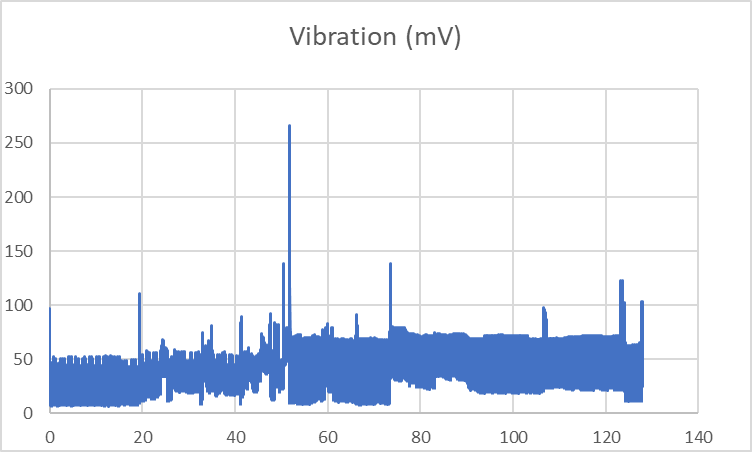  Time (h) |

**Fig. S5: Log of a) temperature, b) humidity and c) vibration during the shipment. Vibration values were represented as raw values (mV) where up to 50 mV was the baseline (no vibration) and more than 100 mV was from a mild shock.**

| 2D control  freshly prepared  freshly prepared control  Pre-prepared  Pre-prepared control | 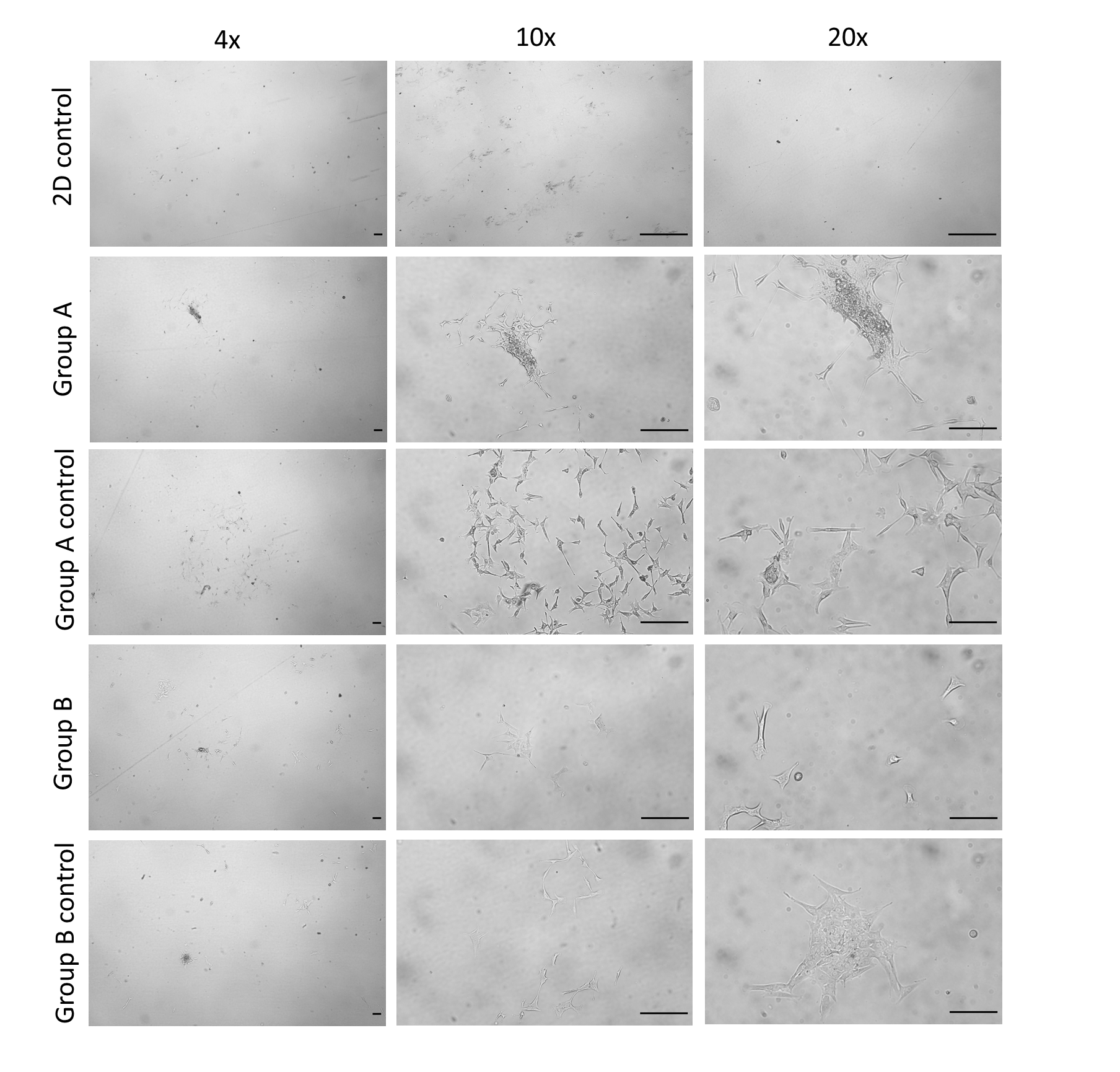 |
| --- | --- |
| **Fig. S6: A549 cells after 48 h of recovery from alginate hydrogel beads shipped after freshly prepared or from 1-week prep-prepared, with their controls. Scale bare for 4x magnification is 500 µm and for 10x, and 20x is 100 µm** | |

***Arduino code used to assemble the data logger with Arduino IDE:***

| /*  SD card datalogger  The circuit:  analog sensors on analog ins 0, 1, and 2  SD card attached to SPI bus as follows:  ** MOSI - pin 11  ** MISO - pin 12  ** CLK - pin 13  ** CS - pin 4 (for MKRZero SD: SDCARD_SS_PIN)  */  #include <SPI.h>  #include <SD.h>  const int chipSelect = 4;  int vib = 0;  #include "DHT.h"  #define DHTPIN 2 // Digital pin connected to the DHT sensor  #define DHTTYPE DHT11 // DHT 11  DHT dht(DHTPIN, DHTTYPE);  void setup() {  // Open serial communications and wait for port to open:  Serial.begin(9600);  while (!Serial) {  ; // wait for serial port to connect. Needed for native USB port only  }  Serial.print("Initializing SD card...");  // see if the card is present and can be initialized:  if (!SD.begin(chipSelect)) {  Serial.println("Card failed, or not present");  // don't do anything more:  // while (1);  }  Serial.println("card initialized.");  Serial.println(F("DHTxx test!"));  dht.begin();  }  void loop() {  vib = analogRead(A0);  // Wait one second between measurements.  delay(1000);  // Reading humidity  float h = dht.readHumidity();  // Read temperature as Celsius (the default)  float t = dht.readTemperature();  // Read temperature as Fahrenheit (isFahrenheit = true)  float f = dht.readTemperature(true);  // Check if any reads failed and exit early (to try again).  // if (isnan(h) \|\| isnan(t) \|\| isnan(f)) {  // Serial.println(F("Failed to read from DHT sensor!"));  // return;  // }  // Compute heat index in Fahrenheit (the default)  float hif = dht.computeHeatIndex(f, h);  // Compute heat index in Celsius (isFahreheit = false)  float hic = dht.computeHeatIndex(t, h, false);  Serial.print(F(" Humidity: "));  Serial.print(h);  Serial.print(F("% Temperature: "));  Serial.print(t);  Serial.print(F("C "));  Serial.print(f);  Serial.print(F("F Heat index: "));  Serial.print(hic);  Serial.print(F("C "));  Serial.print(hif);  Serial.println(F("F"));  Serial.println(vib);  //SD  // open the file. note that only one file can be open at a time,  // so you have to close this one before opening another.  File dataFile = SD.open("SHIP.TXT", FILE_WRITE);  // if the file is available, write to it:  if (dataFile) {  dataFile.print(millis());  dataFile.print(",");  dataFile.print(h);  dataFile.print(",");  dataFile.print(t);  dataFile.print(",");  dataFile.println(vib);  dataFile.close();  // print to the serial port too:  Serial.print(millis());  Serial.print(",");  Serial.print(h);  Serial.print(",");  Serial.print(t);  Serial.print(",");  Serial.println(vib);  }  // if the file isn't open, pop up an error:  else {  Serial.println("error opening SHIP.TXT");  }  } |
| --- |
